# Deep Learning and Histogram-Based Grain Size Analysis of Images

**DOI:** 10.3390/s24154923

**Published:** 2024-07-30

**Authors:** Wei Wei, Xiaohong Xu, Guangming Hu, Yanlin Shao, Qing Wang

**Affiliations:** School of Geosciences, Yangtze University, Wuhan 430100, China; weiwei8248@yangtzeu.edu.cn (W.W.);

**Keywords:** grain size analysis, deep leaning, histogram layer, grain size cumulative curve, simulation experiment

## Abstract

Grain size analysis is used to study grain size and distribution. It is a critical indicator in sedimentary simulation experiments (SSEs), which aids in understanding hydrodynamic conditions and identifying the features of sedimentary environments. Existing methods for grain size analysis based on images primarily focus on scenarios where grain edges are distinct or grain arrangements are regular. However, these methods are not suitable for images from SSEs. We proposed a deep learning model incorporating histogram layers for the analysis of SSE images with fuzzy grain edges and irregular arrangements. Firstly, ResNet18 was used to extract features from SSE images. These features were then input into the histogram layer to obtain local histogram features, which were concatenated to form comprehensive histogram features for the entire image. Finally, the histogram features were connected to a fully connected layer to estimate the grain size corresponding to the cumulative volume percentage. In addition, an applied workflow was developed. The results demonstrate that the proposed method achieved higher accuracy than the eight other models and was highly consistent with manual results in practice. The proposed method enhances the efficiency and accuracy of grain size analysis for images with irregular grain distribution and improves the quantification and automation of grain size analysis in SSEs. It can also be applied for grain size analysis in fields such as soil and geotechnical engineering.

## 1. Introduction

Sedimentary physical simulation is an important method used in sedimentology, simulating sedimentary processes in natural environments through physical experiments. Sedimentary simulation experiments (SSEs) can help to analyze and understand the transport, deposition, and distribution of sediments [1,2,3], and the SSE results can be used to guide practical engineering projects, such as river management, coastal protection, and oil and gas exploration. Grain size analysis is a critical indicator in simulation experiments, revealing the hydrodynamic characteristics of sedimentary processes and playing a crucial role in identifying sedimentary microfacies [4,5]. Traditional laboratory methods for grain size analysis, such as counting, sieving, sedimentation, and laser diffraction, are time consuming, costly, and only suitable for localized analysis [6]. With the development of computer vision, methods for analyzing grain size based on images have become a new research focus due to their speed, low cost, high precision, and wide coverage [7]. These methods significantly improve the precision and efficiency of data processing in SSEs.

Methods for analyzing grain size based on images can be primarily categorized into two groups: edge detection-based and texture-based approaches [8,9]. In edge detection-based techniques, the grains edges in images are first identified, following which geometric information is extracted, such as shapes and grain sizes. Common techniques for edge detection involve image segmentation, thresholding, and watershed methods [10,11,12]. These edge detection-based techniques require the grain edges in images to be distinctly visible. However, most grains are of small size, and their edges are not clearly visible in images from SSEs, rendering edge-based techniques unsuitable.

Texture-based approaches for analyzing grain size can overcome the constraints of edge detection-based techniques. These approaches primarily rely on differences in grayscale values between image pixels. Texture-based approaches for grain size analysis fall into two categories: statistical and multi-scale transformation methods. In statistical methods, the spatial relationships of grayscale values between image pixels are used to identify texture features. Commonly used statistical techniques include histogram and binarization, gray-level co-occurrence matrix [13], texture spectrum statistics [6,14], autocorrelation function [15,16], and semivariogram [17]. These statistical methods are intuitive, highly flexible, and suitable for capturing local texture features, but they are sensitive to noise and require complex parameter design and substantial expert knowledge. Multi-scale transformation methods for grain size analysis involve transforming images across multiple scales, mapping images from their original space to a new feature space, and establishing a relationship between the new space and grain size distribution. Common multi-scale transformation methods include Fourier transform, wavelet transform [18,19,20], and empirical mode decomposition [21]. The core technologies of multi-scale transformation methods are signal processing algorithms, which excel at separating and identifying texture information with different frequencies or scales. However, multi-scale transformation methods require a certain regularity in the grains arrangement within images, i.e., well-sorted grains.

In recent years, deep learning approaches have effectively overcome the challenge of parameter adjustment compared with traditional image-based methods. They eliminate complex transformation formulas, reduce the impact of scale variations, and have significantly enhanced recognition efficiency and accuracy. Deep learning methods have been widely applied for grain size analysis in edge detection-based scenarios, including assessing beach gravel sizes in high-definition images [22], UAV-based riverbed gravel analysis, and grain size analysis from thin-section digital images [23]. Due to their high efficiency and automated edge extraction capabilities, deep learning methods reduce manual costs and greatly enhance the efficacy of image-based grain size analysis. However, applications of deep learning in texture-based grain size analysis remain limited. Buscombe [8] used standard convolutional neural networks (CNNs) to analyze image grain size. While traditional CNNs are powerful tools for extracting features such as color and shape, their performance in texture-based classification tasks remains limited [24]. To overcome this limitation, many researchers have proposed new architectures based on existing networks to extract texture features from images, such as DeepTen [25], DEPNet [26], DSRNet [27], CLASSNet [28], FENet [29], and histogram [30,31]. Although there has been extensive research combining deep learning with texture analysis, relatively few studies have focused on its application in grain size analysis.

In this study, we combine deep learning networks with the histogram algorithm. Through extracting local features, we effectively improve the accuracy of grain size analysis for images with fuzzy boundaries and poorly sorted grains. The main contributions of this paper are as follows:

(1) Developing the Sedihist model to estimate grain size corresponding to cumulative volume percentage. This model combines deep learning with the histogram layer to enhance the accuracy of grain size analysis for images with irregular grain arrangements based on texture.
(2) Proposing a practical workflow for grain size in practice, encompassing data acquisition, data processing, data analysis, and application.
(3) Providing optimal parameters and image resolution for grain size analysis in SSE, offering valuable references for model applications.

The remainder of this paper is structured as follows: Section 2 describes the data acquisition process; Section 3 introduces our research methodology. A detailed design and experimental results are presented in Section 4, including comparisons with eight other models. The discussion and application are covered in Section 5. Finally, Section 6 summarizes the study’s findings.

## 2. Data Acquisition and Preprocess

**Figure 1:**
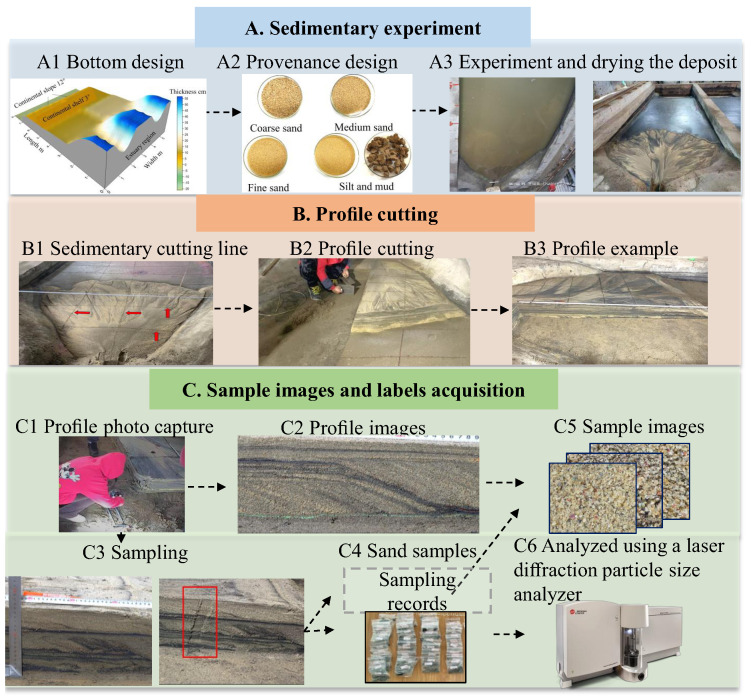
Flowchart of data and labels acquisition.

The data used in this study were sourced from an SSE conducted at the flume simulation laboratory of the Jingzhou campus of Yangtze University in collaboration with the China National Petroleum Corporation (CNPC). We simulated geological environments and terrestrial clastic deposits through designing flume beds with varying slopes and sand grains of different sizes (see Figure 1(A1,A2)). Different climatic conditions were simulated with various water flow modes. After the experiment, the sedimentary body was air-dried for about a month (see Figure 1(A3)) before proceeding to the subsequent data collection stage.

### 2.1. Data Acquisition

Data acquisition for this study comprised two tasks: on-site data collection and indoor data processing. The on-site data collection involved cutting the dried sedimentary body to understand its internal structure and sand distribution patterns. The cuts were made along pre-planned cutting routes (as shown in Figure 1(B1)) using specialized cutting tools (see Figure 1(B2)). After each cut, the exposed profile was documented, and data collection was performed on the profile. First, high-resolution images of the profiles were captured using a Canon 9D camera (as shown in Figure 1(B3)). The camera was mounted on a gimbal and placed on a stable rail system (Figure 1(C1)) to ensure consistency in resolution and shooting angle (Figure 1(C2)). Following this, rulers were placed above and beside the sampling sites to record detailed location information (Figure 1(C3)). The samples were taken by scraping the 23 mm wide and high surface layer of the profile using a 23 mm wide scraper. The samples were then placed in sample bags (Figure 1(C4)). This data collection process was repeated for subsequent cuts along the planned cutting routes until all were completed.

**Figure 2:**
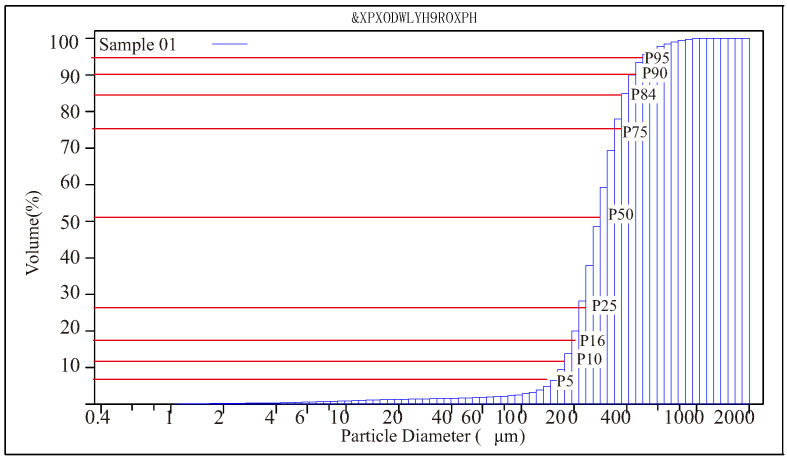
Cumulative curve of one sample based on the LPSA and the positions of the nine labels used in this study.

Indoor data processing involved image cropping and grain size measurement. Image cropping was performed based on records using the ArcGIS10.6 software (Figure 1(C5)). To measure grain size, the dried samples were analyzed using a Beckman Coulter LS13320 Laser Particle Size Analyzer (LPSA, Beckman Coulter Life Sciences, Indianapolis, IN, USA) (as shown in Figure 1(C6)). The measurement range of this device is 0.4–2000 μm. The device determines the volume distribution of grains through laser diffraction and Polarization Intensity Differential Scattering (PIDS) technology and then performs cumulative calculations to obtain the volume cumulative distribution. Figure 2 shows the positions of the nine cumulative percentages on the cumulative curve: 0.05, 0.1, 0.16, 0.25, 0.5, 0.75, 0.84, 0.9, and 0.95. The corresponding grain sizes are the labels used in this study.

In total, 500 samples were collected, including 500 sample images and corresponding laser measurements. The cropped images were 224 × 224 pixels with a resolution of 0.1 mm. The grain size of the samples was less than 2 mm.

### 2.2. Data Preprocessing

Due to the variations in image acquisition time, variations in lighting can cause changes in image color. To minimize external interference with texture analysis, color normalization preprocessing was required. Grayscale image conversion was performed to convert RGB channels into grayscale using the method referenced in television broadcasting standard Rec.601 [32], which was established by the International Telecommunication Union (ITU-R). The detailed formula is presented in Equation (1):

(1) Y=0.299R+0.587G+0.114B

where *Y* represents the grayscale value of the transformed image, and *R*, *G*, and *B* correspond to the red, green, and blue components of the original image, respectively. To enhance data diversity and improve the model’s robustness to the spatial positioning of images, we applied two image augmentation methods: vertical flipping and horizontal flipping, each with an application probability of 0.5, following the approach described by Bihani [33].

## 3. Methods

In recent years, a variety of outstanding network models have emerged in the field of deep learning, such as DenseNet [34], EfficientNet [35], ViT [36], and DeiT [37]. These have achieved significant results in various domains and tasks. In contrast, Residual Network (ResNet) is one of the classic models for mitigating the vanishing gradient problem, thus maintaining network stability and training efficiency. In practical applications, ResNet remains popular due to its simple and stable structure, ease of training and tuning, and strong performance in various image processing tasks [38]. However, traditional deep learning networks have certain limitations in extracting texture features. Typically, as the network depth increases, the pooling operations in the upper layers can lead to a loss of local texture features, thereby affecting grain size analysis performance [24]. The proposed model employed the lightweight ResNet18 network as the backbone, integrating the concept of local histograms to extract local texture features. This model is suitable for grain size analysis with limited data and poor grain sorting.

### 3.1. Histogram Principles

#### 3.1.1. Histogram

The histogram is commonly used in computer vision and machine learning to capture statistical information of images or feature maps, offering unique advantages in handling image translation, rotation, and scaling [39]. Although a step function is the optimal mathematical expression for representing histograms, its application in deep learning networks is limited owing to its derivative being zero in most regions [40]. Researchers have proposed various improvements, such as ReLU, gate activation function [31], Gaussian kernel function [40], and linear basis function [41]. In this study, we used the Gaussian radial basis function (RBF) as the activation function for the histogram layer. The Gaussian RBF offers smooth and flexible characteristics, and its non-zero derivative ensures effective gradient propagation through network layers. In addition, the smoothness of the Gaussian function helps to reduce quantization errors, thereby enhancing the overall accuracy and stability of the model. The definition is described in Equation (2):

(2) $$\varphi \left(x\right)=e^{-\frac{{\left(x-\mu_{k}\right)}^{2}}{2\sigma^{2}}}$$

where φ(x) represents the value of the RBF, $\mu_{k}$ is the center point, and σ is the standard deviation indicating the degree of spread and localization of the RBF, which is a parameter used to control the bandwidth of the function.

**Figure 3:**
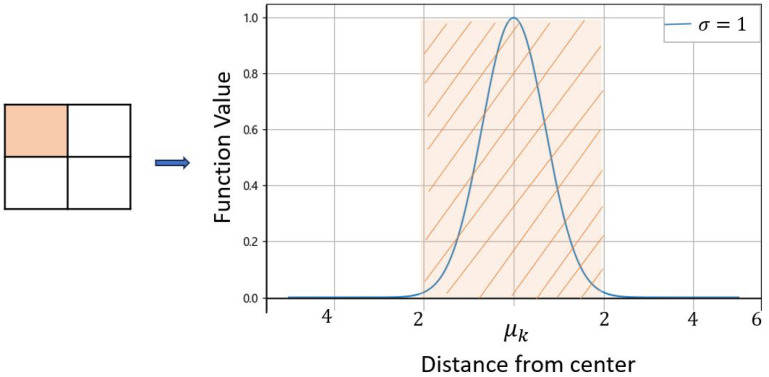
Schematic diagram of Gaussian RBF.

Figure 3 shows that the Gaussian RBF reaches its maximum value of one at the input point closest to the center and rapidly decreases to near zero as the distance increases. In histogram analysis, $\mu_{k}$ can be set as the center of the histogram bin, and σ can be set as the bin width to more accurately simulate the characteristics of the histogram.

#### 3.1.2. Histogram Layer Algorithm

Global histograms can effectively reflect overall texture variations in an image, but they often overlook the details of local textures. In this study, local histograms were employed to capture subtle differences in the images. The Gaussian activation values were calculated based on the distance between the feature values of the local regions and the center of each histogram bin. The local histogram method not only captures local statistical information but also allows the adjustment of the standard deviation of the Gaussian function to control the bin width, thereby tuning the model’s sensitivity.

**Figure 4:**
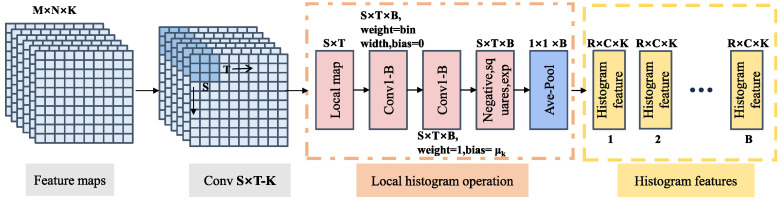
Structure of the histogram layer in the Sedihist model.

The process of generating local histograms is illustrated in Figure 4: First, a sliding window of size S×T was applied to the original image (size M×N). A convolution operation was performed with a 1×1×B kernel, where B is the number of histogram bins. For the bth bin, the kernel weights were set to 1, and the bias was set to the bin center $\mu_{k,b}$. Then, another 1×1×B kernel was used for convolution with kernel weights corresponding to the bin width $w_{k,b}$ and bias set to 0. This was followed by negation (taking the negative value), squaring, and exponential operations, and the histogram features of the local region were accumulated using average pooling. Finally, normalization was performed (Equation (3)). In this way, B histogram features vectors were generated for each local region, S×T. As the window slided, the histogram feature values of the next local region were generated. Calculating histogram features was conducted independently in each channel, such that the original image, M×N×K, passed through the histogram layer and output B feature maps of size r×c×K.

(3) $$Y\left(k\right)=\frac{1}{ST}\sum_{i=1}^{S}\sum_{j=1}^{T}e^{-w^{2}{\left(x_{ij}-\mu_{k}\right)}^{2}}$$

where $\mu_{k}$ represents the center of the histogram bin, and *w* denotes bin width.

#### 3.1.3. Training Histogram Layer Parameter

In the histogram layer, parameter learning and updating were performed through backpropagation. The backpropagation algorithm of the histogram layer was based on the gradient computation of each histogram bin. Specifically, the gradient with respect to each feature map was calculated by the weighted sum of the gradients with respect to each bin. The weights were the derivatives of the Gaussian activation values corresponding to each element. This parameter learning method ensured that the changes in the loss function could be effectively updated according to the contribution of each feature element to the histogram bins. The backpropagation methods for the relevant centers and widths were calculated using Formulas (4) and (5) as shown below:

(4) $$\frac{\partial Y(k)}{\partial \mu_{k}}=\frac{1}{MN}\sum_{i=1}^{M}\sum_{j=1}^{N}2w^{2}\left(x_{ij}-\mu_{k}\right)e^{-w^{2}{\left(x_{ij}-\mu_{k}\right)}^{2}}$$

(5) $$\frac{\partial Y(k)}{\partial w}=\frac{1}{MN}\sum_{i=1}^{M}\sum_{j=1}^{N}-2w{\left(x_{ij}-\mu_{k}\right)}^{2}e^{-w^{2}{\left(x_{ij}-\mu_{k}\right)}^{2}}$$

### 3.2. Sedihist

**Figure 5:**
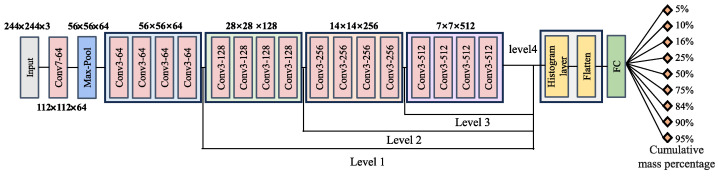
Framework of the Sedihist model.

In this study, we propose the sedimentary histogram deep learning model (Sedihist) in order to address the problem of blurred grain boundary images and poor sorting in SSEs. The model employs the lightweight ResNet18 for feature extraction. The features generated by this network are processed through a histogram layer to obtain local texture information. Finally, all histogram features are flattened and passed through a fully connected layer to achieve end-to-end cumulative grain size percentage values. The specific architecture of the Sedihist model is illustrated in Figure 5. Figure 5 shows four combinations where the histogram layer is placed after the first through fourth residual blocks in ResNet18, corresponding to levels 1–4.

## 4. Results

### 4.1. Experimental Design and Parameter Settings

The experimental content included three parts.

Part 1: In the Sedihist model, features are extracted by ResNet18 with its four residual blocks capturing different feature levels. Consequently, introducing histogram layers at various positions within the network leads to variations in input features, thereby affecting the model’s overall accuracy. Moreover, the local window size and number of bins in the histogram layer directly determine the granularity of feature extraction, critically impacting the model’s accuracy. Therefore, we systematically conducted detailed experiments to determine the optimal position (as shown in Figure 5, levels 1–4), window size (as shown in Figure 4, S×T), and number of bins (as shown in Figure 4, B) for the histogram layer.

Part 2: To evaluate accuracy of the Sedihist model, the experiment included a total of 500 samples, which were randomly divided into 450 training samples and 50 test samples. As the measurements obtained from the LPSA were highly accurate and considered the ground truth values for the samples, the model accuracy was evaluated by comparing its estimated values with the analyzer measurements. The model parameters were set as follows: the optimal parameters determined in Part 1 were referenced. The initialization parameters for the bin centers and bin widths of the histogram layer were generated randomly. The input size of the images was 224×224, and color normalization and data augmentation were performed. The loss function was Smooth L1, and the optimization algorithm was stochastic gradient descent. The training cycle was set to 100 epochs with a batch size of 8 and a learning rate of 0.0001.

Part 3: To further validate the model’s effectiveness, the results of this model were compared with those of eight other commonly used models. These models include the Wavelet analysis [18], SediNet [8], DeepTen [25], VGG [42], VGG_hist (integration of VGG and the histogram layer), ResNet18 [38], ResNet50 [38], and ResNet50_hist (integration of ResNet50 and the histogram layer). Except for the wavelet method, all other models are deep learning network models, and the training and test samples were the same as those used for the Sedihist model. The initial parameters for the wavelet method were set to r = 0.05, m = 10, x = 0.1, and f = 0. The main parameters for the SediNet model were grayscale = true, scale = false, and dropout = 0.25. The parameters for DeepTen, VGG, ResNet18, and ResNet50 were kept at their defaults with the output layer modified to correspond to the cumulative percentage grain size values. A histogram layer was added to VGG_hist and ResNet50_hist before the fully connected layer.

The model’s implementation was based on Python 3.6 and Pytorch 2.1.2, running on a system with 32 GB of RAM (Kingston Technology, Fremont, CA, USA) and an RTX 3080Ti GPU (NVIDIA Corporate, Santa Clara, CA, USA).

### 4.2. Evaluation Metrics

In this study, the evaluation metrics were normalized root mean square error (NRMSE), mean absolute percentage error (MAPE), and Chi-squared distance ($\chi^{2}$ distance). Each metric possesses unique advantages and assesses the model’s performance from different perspectives. NRMSE combines bias and variance, focusing on absolute error. MAPE calculates relative error, balancing data of varying magnitudes. Chi-squared distance addresses differences in distribution, making it suitable for comparing predicted and actual cumulative grain size distributions. Combining these three evaluation metrics provides comprehensive insight into the model’s performance. A detailed explanation of the three evaluation metrics is as follows.

NRMSE is a normalized metric that facilitates comparisons between different datasets. It is calculated by normalizing the root mean square error to the average actual grain size of all samples at each percentile and is expressed as a percentage (Equation (6)). MAPE is computed by taking the absolute difference between the actual and estimated values at each percentile, dividing by the actual value, and then averaging these ratios, which is typically expressed as a percentage (Equation (8)). NRMSE and MAPE were calculated separately for the grain size values corresponding to the nine cumulative percentages. The average of these nine percentiles was taken to obtain the overall NRMSE and MAPE values, which served as the overall accuracy metrics of the model (Equations (7) and (9)).

(6) $$NRMSE_{ji}=\frac{\sqrt{\frac{1}{n}\sum_{i=1}^{n}{\left(y_{i}-{\hat{y}}_{i}\right)}^{2}}}{\frac{1}{n}\sum_{i=1}^{n}y_{i}}\times 100\%,$$

(7) $$NRMSE=\frac{1}{p}\sum_{j=1}^{p}NRMSE_{p},$$

(8) $$MA\;PE_{ji}=\frac{1}{n}\sum_{i=1}^{n}\left|\frac{y_{i}-{\hat{y}}_{2}}{y_{i}}\right|\times 100\%,$$

(9) $$MA\;PE=\frac{1}{p}\sum_{j=1}^{p}MA\;PE_{p},$$

where i=1,2…n with n being the number of test samples; and j=1,2…p,p=9 corresponding to the nine cumulative percentages, and $y_{i}$ and ${\hat{y}}_{i}$ being the measured and estimated values of the grain size for the *i*-th sample at a specific cumulative percentage, respectively.

Chi-squared distance was used to compare the similarity between two probability distributions. It measures the difference between two distributions by calculating the sum of the squared differences between their elements, reflecting their similarity. The calculation is as shown in Equation (10):

(10) $${\chi^{2}}_{ij}=\sum_{j=1}^{p}\frac{{\left(y_{j}-\hat{y_{j}}\right)}^{2}}{\left(y_{j}+{\hat{y}}_{j}\right)},$$

where ${\chi^{2}}_{ij}$ is the Chi-squared distance for the *i*-th sample, and $y_{j}$ and ${\hat{y}}_{j}$ are the measured and estimated grain size values for the *j*-th cumulative percentage of the *i*-th sample, respectively.

### 4.3. Experimental Results

#### 4.3.1. Determining the Optimal Parameters

Table 1 shows the accuracy evaluation of the models based on different parameter combinations in terms of histogram position and window size. The results indicate that models with histogram layers positioned at level 2 to level 4 have higher accuracy than level 1. This is because levels 2 to 4 are situated in deeper network layers, enabling the extraction of more extensive mid-to-high level features [24,30]. The overall accuracy differences among levels 2, 3, and 4 were minimal, demonstrating the robustness of the histogram layer. The accuracy of level 3 was slightly higher than levels 2 and 4, and thus, the optimal histogram layer position was selected as level 3. Regarding window size, models with medium-sized windows achieved slightly higher accuracy than those with smaller or larger windows, which is likely because medium-sized windows better balance the capturing of local and global information [43]. Therefore, the optimal window size was chosen as 10×10.

**Figure 6:**
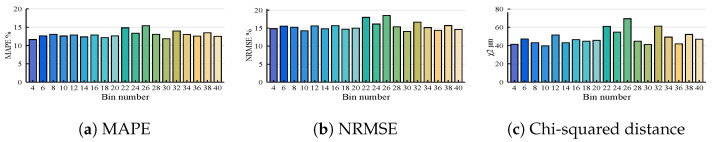
Accuracy evaluation with different numbers of bins.

Concerning the number of bins, Figure 6 illustrates the accuracy evaluation of models with different bin numbers. The results indicate that within a range of 4 to 40 bins, the Sedihist model’s accuracy did not significantly change, suggesting that ResNet18 may have already effectively extracted the key features. This finding implied that variations in the number of bins have a limited impact on the model’s final performance. In practice, increasing the number of bins mainly affects computational efficiency rather than accuracy. When designing a model, it is necessary to balance the number of histogram bins to achieve the optimal trade-off between efficiency and effectiveness [30]. In this study, we chose a smaller number of bins, specifically four, to reduce complexity.

#### 4.3.2. Visualizing Model Results

Visualizing the features extracted by CNN is important for enhancing the interpretability of models. Gradient-weighted Class Activation Mapping (Grad-CAM) generates a two-dimensional heatmap using feature maps from the last convolutional layer of a CNN, indicating the importance of different regions in the input image for a specific category [44]. This technique computes the partial derivatives of the estimated value with respect to each channel, interpreting these derivatives as the importance scores for each channel. These scores are then multiplied by the channels to obtain each channel’s contribution to the predicted value.

**Figure 7:**
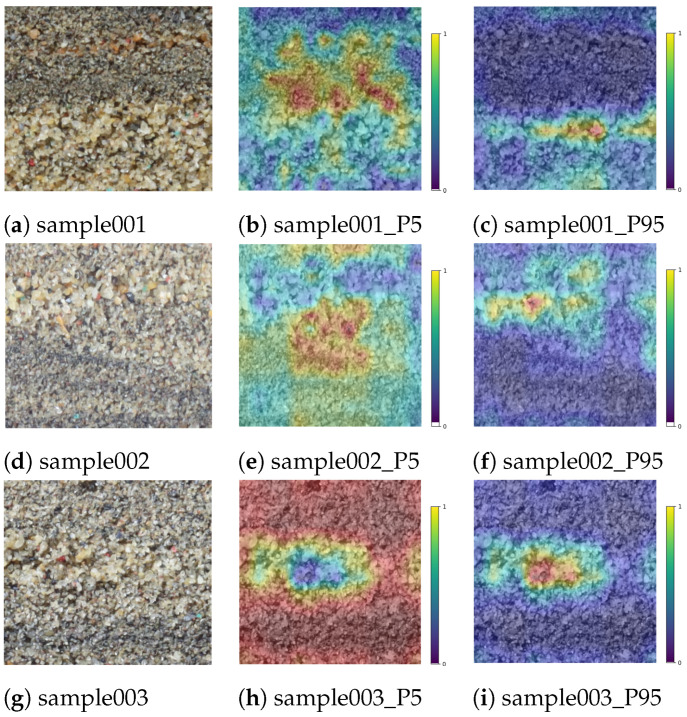
Grad-CAM heatmaps of three test images, showing the heapmaps at the 5th and 95th cumulative percentiles.

In this study, we generated Grad-CAM heatmaps by calculating the product of the average gradient across each channel of the feature maps and the channels themselves. We selected three samples and displayed the Grad-CAMs for the 5th and 95th percentiles of the cumulative distribution, as shown in the Figure 7. Through visually inspecting these three sample images, the distribution of grains of different sizes can be roughly discerned. For instance, in the first sample, fine grains are mainly distributed in the upper part of the image, while coarse grains are concentrated in the lower part. The Grad-CAM of the 5th percentile cumulative percentage shows stronger activations in the upper part of the image, whereas the Grad-CAM of the 95th percentile shows stronger activations in the lower part. The other two samples yielded similar conclusions.

These results indicate that the CAM of the 5th percentile primarily focuses on fine grains, while the CAM of the 95th percentile primarily focuses on coarse grains, which is consistent with the theoretical expectations of cumulative percentiles. This not only aids in better understanding the model’s mechanisms but also validates the effectiveness of our method.

#### 4.3.3. Accuracy Evaluation

**Figure 8:**
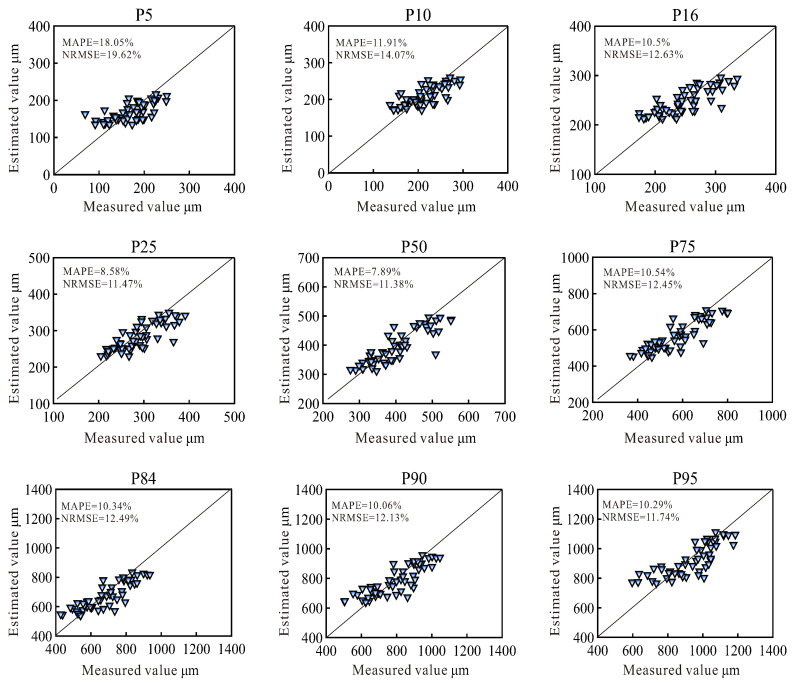
Comparison of estimated values from the Sedihist model and measured values from LPSA.

Figure 8 compares between the estimated grain size corresponding to the nine cumulative percentages using the Sedihist model and the measurements from the LPSA. The results indicate high concordance between the Sedihist estimations and the analyzer measurements. The accuracy range for NRMSE was 11.38% to 19.62%, and for MAPE, it was 7.89% to 18.05%. Compared with previous research results, indicating an MAPE range of 24.5–45% [8], this model demonstrates significantly improved accuracy. There were significant differences in accuracy across different cumulative percentages. The highest accuracy was at the 50% cumulative percentage with NRMSE and MAPE values of 11.38% and 7.89%, respectively. Conversely, the lowest estimation accuracy was at the 5th percentile of cumulative distribution, with NRMSE and MAPE values of 19.62% and 18.05%, respectively. This indicated that the estimation accuracy for median grain sizes was the highest, and there was a larger deviation in the estimating smaller grain sizes.

#### 4.3.4. Comparison of Different Model Accuracy

**Figure 9:**
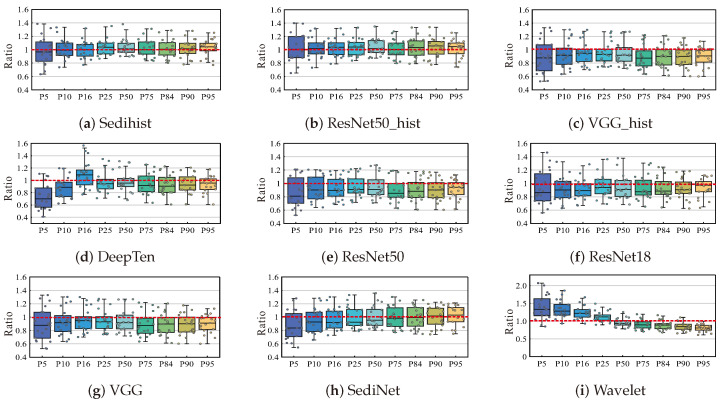
Box plot analysis comparing the ratios of values measured by the LPSA to those estimated by models. The points in the box plot represent the ratios for test samples.

Table 2 compares the accuracy, parameter count, and inference time between the Sedihist model and the eight other commonly used models. In addition, we calculated the ratio of the values measured by the LPSA to estimate the values of different models to analyze the accuracy. A ratio closer to one indicated that the estimated values were closer to the actual values, a ratio lower than one indicated that the estimated values were greater than the measured values, and a ratio greater than one indicated that the estimated value was less than the measured values. The results are shown in Figure 9.

The results indicated that the Sedihist model performed the best in terms of MAPE, NRMSE, and Chi-squared distance, achieving values of 10.91%, 13.11%, and 32.2 μm, respectively. In addition, the Sedihist model demonstrated high stability in grain size analysis at different cumulative percentages compared with other methods (see Figure 9) with ratio results mostly close to 1. The model tends to overestimate with smaller cumulative percentages and slightly underestimate with the 95th percentile of cumulative distribution. The ResNet50_hist model showed similarly high accuracy but with more parameters and greater complexity. Of the nine models, the wavelet model has the worst accuracy, with MAPE, NRMSE, and Chi-squared distance values of 19.21%, 23.15%, and 101.6 μm, respectively. Figure 9 also shows that the wavelet method is the most unstable with large errors in grain size estimations at both ends of the cumulative percentages. This instability is related to the wavelet analysis algorithm, which is unsuitable for scenarios with poor grain sorting [18].

The SediNet model used only a traditional CNN. Although this model has a significant advantage in terms of parameter count compared with the other eight models, traditional CNNs are limited in texture recognition, resulting in lower accuracy compared with the Sedihist model. Among the nine models, three are base models (ResNet18, ResNet50, and VGG) and three corresponding versions are with combined with a histogram layer (Sedihist, ResNet50_hist, and VGG_hist, respectively). Both Table 2 and Figure 9 show that the models combined with a histogram layer have higher estimation accuracy than the base models alone, proving the effectiveness of the histogram layer in improving texture recognition accuracy. The DeepTen model showed moderate accuracy in this experiment, possibly because it performed well in handling local features but had shortcomings in integrating global features, leading to poor consistency in grain size estimations across different cumulative percentages.

**Figure 10:**
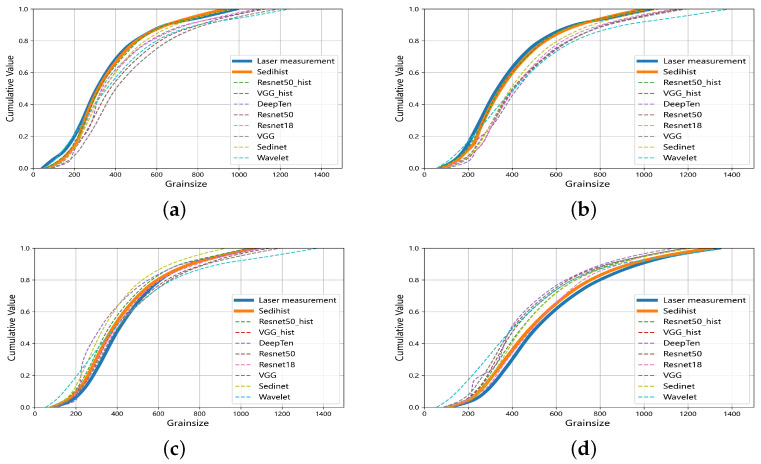
Comparison of cumulative curves for four samples using different models. The mean grain sizes of four samples (**a**–**d**) are 253 μm, 334 μm, 408 μm and 511 μm, respectively. The thick yellow lines represent cumulative curves based on the Sedihist model, and the thick blue lines represent measurements from the LPSA.

Figure 10 shows the estimated cumulative curves of the nine models for four samples with different mean grain sizes. These cumulative curves were interpolated using the piecewise cubic Hermite interpolating polynomial (PCHIP) method. From Figure 10a to Figure 10d, as the average grain sizes of the samples gradually increase, the model estimations change accordingly: initially, the estimated values are larger than the measured values. Then, they approach the measured values and, finally, the estimated values become smaller than the measured values. In other words, when the mean grain size is smaller, the model tends to overestimate the value; when the average grain size is larger, the model tends to underestimate the value. Overall, the cumulative curves estimated by the Sedihist model show a high degree of agreement with measurements from the LPSA.

## 5. Discussion

The Sedihist model effectively predicts the distribution characteristics of grain sizes in images. However, the model’s accuracy is influenced by the textural differences presented by grain of varying sizes and colors. Additionally, different image resolutions affect the degree of detail captured in images. In this section, we discuss the impacts of sample size, color, and image resolution on model accuracy. Furthermore, we apply our model in practical scenarios to verify its generalizability.

### 5.1. Sensitivity of Sample Size and Color

To thoroughly understand the model’s sensitivity to grain size, we selected 10 samples with an average grain size of less than 100 μm and 10 samples with an average grain size of more than 1000 μm, dividing them into two groups. The estimation accuracy for these two groups is shown in Table 3.

Table 3 shows that the MAPE, NRMSE, and Chi-square distance for large-sized grains are significantly lower than those for small-sized grains. This phenomenon is mainly due to the more prominent texture and edge features of large-sized grains in images, which the Sedihist model can capture more efficiently. However, for smaller-sized grains, the finer features in images are more susceptible to noise and image resolution effects, posing a greater challenge for the Sedihist model during feature extraction. Although the histogram layer can provide statistical information for local regions, its effectiveness may decrease when handling fine features.

**Figure 11:**
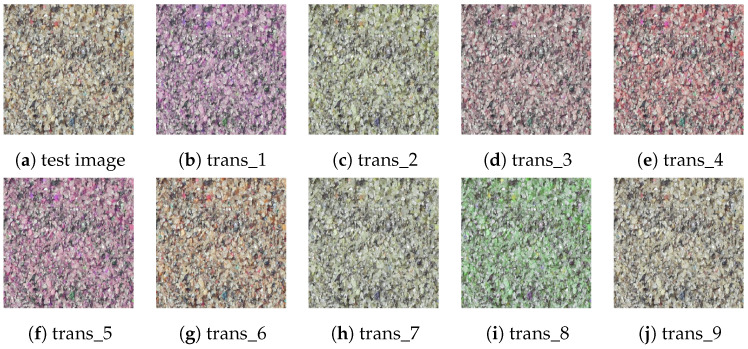
Results of random color transformations on the test image. (**a**) Test image, (**b**–**j**) are nine randomly color-transformed images.

**Figure 12:**
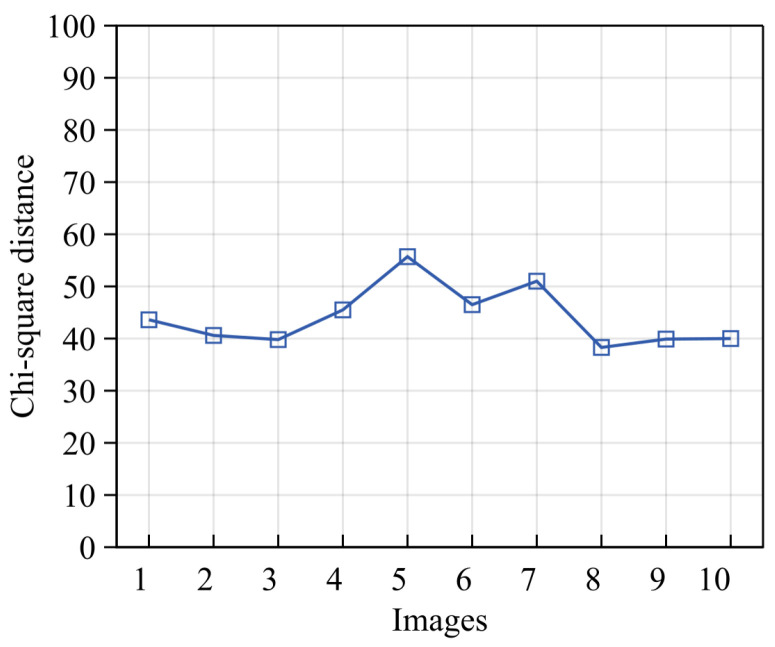
Color sensitivity assessment. Chi-squared distance accuracy evaluation for the 10 images in Figure 11.

Regarding sensitivity to grain color, as the images in this study were all collected in an indoor flume laboratory with controlled ambient lighting, variations in image brightness and contrast due to external lighting conditions can be ignored, and we mainly focus on the impact of color changes caused by lighting or grain composition differences on model accuracy. To simulate grains with different colors, we performed random transformations on the saturation and hue of the test image, with saturation varying between 60% and 140% of the original value, and hue randomly altered within 40% of the original value. Figure 11 shows one sample image with nine transformation results based on the above scheme. Predictions were made for these ten images, and the Chi-squared distance was used to evaluate prediction accuracy (Figure 12). Figure 12 shows that the Chi-squared distances between the predicted values of the nine transformed images and the original image were similar, indicating that the model is insensitive to color. This insensitivity is primarily due to the grayscale normalization applied during the data preprocessing stage, which significantly reduces color interference, allowing the model to focus more on brightness and texture features, thereby enhancing its stability and robustness.

### 5.2. Image Resolution

**Figure 13:**
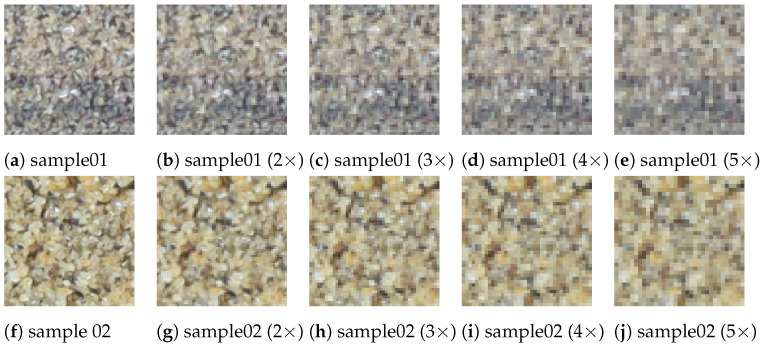
Downsampling results for two sample images: Sample 01 with an average grain size of 278 μm, and Sample 02 with an average grain size of 511 μm. In “n×”, n represents the downsampling factor.

High-resolution images can capture rich texture information. However, increasing the resolution of images results in an exponential increase in the number of photos. Consequently, the computational and time costs for data processing significantly increase. Therefore, it is important to choose an appropriate image resolution in practice. This section discusses the impact of image resolution on analysis accuracy and explores how to select the appropriate resolution based on grain characteristics in SSE scenarios. In this study, the original sample images had a resolution of 0.1 mm. Images with different resolutions were obtained by downsampling the original images, with downsampling factors ranging from 2 to 5, resulting in image resolution of 0.15 mm, 0.2 mm, 0.25 mm, and 0.3 mm. The results for two samples with different mean grain sizes at four downsampling factors are shown in Figure 13. When the image was downsampled by a factor of 4, the blurring of grains became significant, especially for sample 2, which had smaller grain sizes. Table 4 presents an accuracy evaluation of five different resolutions. The results indicate that decreasing image resolution significantly impacts model accuracy. When the downsampling factor was 2 or 3, the model’s estimation error did not change significantly; however, when the downsampling factor reached 4 or higher, the model’s estimation error rapidly increased.

**Figure 14:**
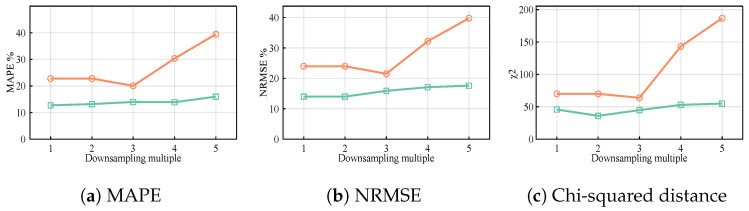
Comparison of estimated values for two sample groups with different grain sizes. Red line represents the group with a smaller average grain size; green line represents the group with a larger mean grain size.

These results were based on all test samples, including small and large grains. To further understand the effect of image resolution on different grain size, we selected two groups of samples using the method outlined in Section 5.1: large-sized grains and small-sized grains. We then evaluated the accuracy for these two groups separately (Figure 14). Figure 14 shows that the orange lines represent small-sized grains, and the green lines represent large-sized grains. The results indicate that decreasing image resolution had a greater impact on the accuracy of small-sized grains than large-sized grains. This might be because the texture information between small grains became blurrier with lower resolution, leading to decreased recognition accuracy.

Overall, image resolution significantly impacts model accuracy. For experiments where the grain size is less than 2 mm, it is recommended that the image resolution should not be less than 0.2 mm. If the grains mainly consist of fine sand and silt or smaller grains, a higher image resolution is necessary, such as 0.05 mm or 0.1 mm. Conversely, if medium-to-coarse sand constitutes a larger proportion of the grains, the image resolution can be appropriately reduced to 0.1 mm or 0.2 mm.

### 5.3. Application

The Sedihist model effectively estimates the grain size of a single image. However, studies must often analyze an entire profile in practice to understand the distribution patterns and depositional modes of sedimentary bodies. This section explores how well the Sedihist model can be applied in practice. We applied this model to an SSE that simulated a delta sedimentary system under a lacustrine transgression background. The experiment included seven sub-experiments, each consisting of one gravity flow and eight traction flows. High-sediment concentration, fast-flowing, short-duration water flows simulated gravity flows caused by episodic floods in nature, while low-sediment concentration, slow-flowing, long-duration water flows simulated traction currents. The rise and fall of water levels in the flume simulated the natural phenomena of lake transgression and regression. The grain diameters were all less than 2 mm, and the image resolution was 0.1 mm.

The traditional manual analysis procedure involves collecting samples from profiles and analyzing the grain size using laboratory equipment. The grain size of the samples are then analyzed by experienced experts to identify grain size information for the entire profile. Manual analysis methods rely on experience, leading to inconsistent labeling.

**Figure 15:**
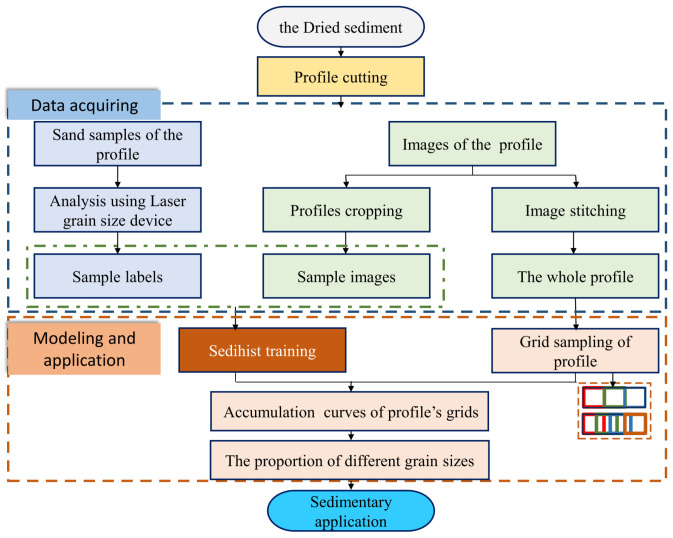
Workflow diagram for grain size estimation of the entire profile.

The process of estimating grain sizes for the entire profile using the Sedihist model is detailed as follows: The image and label acquisition process is shown in Section 2.1. The images obtained served two purposes: cropping images and stitching into a complete profile image. Images need to be collected continuously with an overlap of more than 60% between consecutive images to improve the stitching quality. Grid sampling was performed on the stitched images with sampling parameters including grid size and step size. If the grain size variation across the profile is minor, larger grid sizes and steps can be set; if the variation is significant, smaller grid sizes and steps should be used. After grid sampling, the Sedihist model estimated the grain size corresponding to the nine cumulative percentages for each grid. These estimated values were then interpolated into cumulative curves using the PCHIP method. From these cumulative curves, the volume proportions of medium-to-coarse sand (0.25–2 mm), fine sand (0.1–0.25 mm), and silt and clay (<0.1 mm) for each grid were deduced. Finally, RBF interpolation is used to fit the distribution of different grain sizes across the entire profile. The profile grain size analysis process is illustrated in Figure 15.

**Figure 16:**
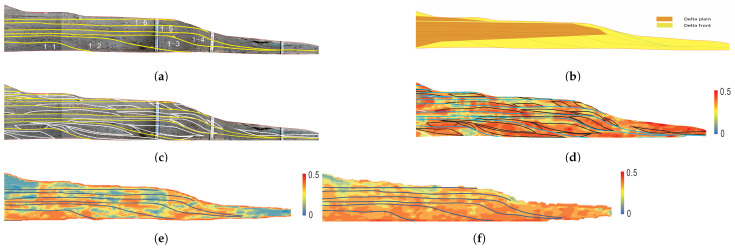
Comparison of the results of manual interpretation and model estimation. (**a**) Original profile image with manually divided sub-experiment areas, sedimentary regions 1–1 to 1–6 correspond to the first to sixth rounds of the experiment, respectively. (**b**) Overall sedimentary environment of the profile. (**c**) Manually identified coarse-grained regions of the profile. The white-circled areas represent the manually identified results. (**d**) Distribution of medium and coarse sand estimated by the Sedihist model; for clarity, the manually identified results are marked in black. (**e**) Distribution of fine sand estimated by the Sedihist model. (**f**) Distribution of silt and clay estimated by the Sedihist model.

Figure 16 presents the estimated results for one of the profiles. The sedimentary sub-facies of this profile include the delta plain and delta front. The lines in Figure 16a represent the boundaries of different sub-experiments (as shown in Figure 16a,b), where sedimentary regions 1–1 to 1–6 correspond to the first to sixth rounds of the experiment, respectively. Figure 16c displays the manually identified distribution of coarse clastic grains within the profile. Figure 16d–f illustrate the distribution of medium-to-coarse sand, fine sand, and silt and clay estimated using the Sedihist model.

In Figure 16d, the red areas indicate a higher proportion of coarse sand, and the black circled areas represent the manually interpreted results. Figure 16d shows that the red areas and the black circled areas have a high degree of agreement. The results indicate that the high coarse sand distribution areas estimated with the Sedihist model closely matched the manually identified coarse clastic distribution. Overall, the Sedihist results agreed with the manual identifications, demonstrating high accuracy and practical value. Additionally, the Sedihist model can quantitatively display the distribution of fine grains, addressing the limitations of human vision in distinguishing fine grains. The proposed method requires collecting samples to train the model parameters, but the estimation process does not require sampling; it directly estimates grain size from profile photos, significantly improving the efficiency and accuracy of grain size analysis.

### 5.4. Limitation and Future Work

Despite the superior accuracy of the Sedihist model compared with the other methods discussed in this paper, there are still some limitations. First, the number of training samples was limited, especially for small-sized grains (diameters less than 100 μm) and large-sized grains (diameters greater than 1000 μm). The limited number of samples was primarily due to the high cost and time-consuming nature of sample collection. The time consumption arises from the need to cut the sediment body to obtain samples, followed by drying to obtain sample labels, while the high cost is due to the expensive LPSA. The data in the table above show lower prediction accuracy for small- and large-sized grain samples, indicating that collecting and labeling a sufficiently large and diverse training set is crucial. In future research, we plan to improve the sample collection process to reduce time and cost, such as using sedimentation methods and related equipment for grain size measurement to establish a large-scale training library and increase the number and diversity of training samples.

The method proposed in this study can only identify sediment grains in images, limiting its scope regarding other object types. Additionally, due to the constraints of the experimental environment and conditions, the measurement range of the LPSA was 0.3–2000 μm, and the maximum grain size identified by this method does not exceed 2 mm. For grains with sizes beyond this range, the recognition accuracy needs further verification.

The estimation error is relatively high when identifying small grains (diameters less than 100 μm), and the performance is poor. This is because the texture between small grains is weak, making recognition difficult. Furthermore, this study included a limited number of silt and clay components, resulting in insufficient training samples. During the data collection phase, the sand samples must be dried, which can lead to the loss of fine grains, further increasing the prediction error. Therefore, to analyze silt or clay grain distribution, future research should increase the number of clay grain samples and explore methods such as multi-scale feature extraction.

## 6. Conclusions

This study focused on grain size analysis based on images from an SSE. The proposed Sedihist model combines the ResNet18 network with the histogram algorithm estimate grain sizes corresponding to cumulative percentages in images. The Sedihist model demonstrated superior accuracy in estimating the grain size distribution when compared with eight other methods. Furthermore, the model was applied to an SSE to estimate the grain size of the entire profile, and the workflow of the model in practice was presented. The results demonstrated high consistency between the model’s estimations and manual estimations. The Sedihist model effectively addresses the challenges of grain size analysis in images with blurred grain edges and poor sorting. This method can also be applied to grain size analyses in related fields such as soil and geotechnical studies. Given that the grain sizes in this experiment were smaller than 2 mm and the experiment type was delta sedimentation, the model’s applicability to grains larger than 2 mm and other types of experiments requires further validation. Future research can focus on these aspects to improve and enhance estimations for extremely small-sized grains.

## Figures and Tables

**Table 1:** Evaluation of model accuracy based on different combinations of histogram layer positions and window sizes.

Location	Window Size	MAPE (%)	NRMSE (%)	Chi-Squared Distance (μm)
level 1	16×16	18.51	19.88	78
	24×24	18.3	21.25	76.1
	32×32	24	25.55	113.4
	40×40	21.56	23.52	94.7
	48×48	21.36	22.03	89.7
	56×56	23.5	24.38	101
level 2	8×8	11.51	13.71	34.8
	12×12	12.66	15.97	52.9
	16×16	13.07	16.78	56.2
	20×20	14.42	17.9	63.9
	24×24	14.15	16.64	53.2
	28×28	15.71	18.46	75.1
level 3	4×4	11.83	15.81	43.8
	6×6	10.9	13.61	33.9
	8×8	11.21	13.67	34.8
	10×10	10.91	13.11	32.2
	12×12	10.86	13.75	34.6
	14×14	11.01	13.82	36
level 4	2×2	13.62	16.18	48.1
	3×3	12.08	15.41	46.3
	4×4	12.65	15.36	45.9
	5×5	12.1	15.41	45.4
	6×6	11	14.05	39
	7×7	11.76	14.48	41.8

**Table 2:** Comparison of accuracy, parameter count and inference time between different models.

Model	MAPE (%)	NRMSE (%)	Chi-Squared Distance (μm)	Parameter Count (M)	Inference Time (ms)
Sedihist	10.91	13.11	32.2	11.27	36.6
ResNet50_hist	11.59	14	36.8	23.44	76.02
SediNet	15.92	17.14	52.1	0.33	1.07
VGG_hist	17.84	19.6	70.9	33.72	109.51
DeepTen	19.99	22.06	81	35.15	114.09
ResNet50	19.91	20.6	81.7	23.53	75.42
ResNet18	19.82	21.02	86.8	11.18	36.31
VGG	19.89	21.07	87.2	33.63	105.22
Wavelet	19.21	23.15	101.6	/	/

**Table 3:** Comparison of estimation accuracy between two groups.

Downsampling Multiple	MAPE (%)	NRMSE (%)	Chi-Squared Distance (μm)
Large-sized grains	12.07	14	45.8
Small-sized grains	22.8	23.98	70.1

**Table 4:** Accuracy evaluation of values estimated by different models.

Downsampling Multiple	MAPE (%)	NRMSE (%)	Chi-Squared Distance (μm)
1	12.07	14.65	44
2	12.86	14.25	40.7
3	11.99	13.39	38.1
4	15.35	16.87	65.2
5	16.8	18.33	67.7

## Data Availability

The data of this experiment used to support the finding of the research are available on github: https://github.com/weizi8248/Sedihist (accessed on 29 July 2024).
